# WikiBuild: A New Online Collaboration Process For Multistakeholder Tool Development and Consensus Building

**DOI:** 10.2196/jmir.1833

**Published:** 2011-12-08

**Authors:** Samir Gupta, Flora T Wan, David Newton, Onil K Bhattacharyya, Mark H Chignell, Sharon E Straus

**Affiliations:** ^1^Department of MedicineUniversity of TorontoTorontoCanada; ^2^The Keenan Research CentreLi Ka Shing Knowledge InstituteSt Michael’s HospitalToronto, ONCanada; ^3^Division of RespirologyDepartment of MedicineSt. Michael’s HospitalToronto, ONCanada; ^4^Department of Family and Community MedicineUniversity of TorontoToronto, ONCanada; ^5^Department of Mechanical and Industrial EngineeringUniversity of TorontoToronto, ONCanada; ^6^Division of GeriatricsDepartment of MedicineSt Michael’s HospitalToronto, ONCanada

**Keywords:** Consensus, focus groups, user-computer interface, Web 2.0, asthma, self-care

## Abstract

**Background:**

Production of media such as patient education tools requires methods that can integrate multiple stakeholder perspectives. Existing consensus techniques are poorly suited to design of visual media, can be expensive and logistically demanding, and are subject to caveats arising from group dynamics such as participant hierarchies.

**Objective:**

Our objective was to develop a method that enables multistakeholder tool building while averting these difficulties.

**Methods:**

We developed a wiki-inspired method and tested this through the collaborative design of an asthma action plan (AAP). In the development stage, we developed the Web-based tool by (1) establishing AAP content and format options, (2) building a Web-based application capable of representing each content and format permutation, (3) testing this tool among stakeholders, and (4) revising this tool based on stakeholder feedback. In the wiki stage, groups of participants used the revised tool in three separate 1-week “wiki” periods during which each group collaboratively authored an AAP by making multiple online selections.

**Results:**

In the development stage, we recruited 16 participants (9/16 male) (4 pulmonologists, 4 primary care physicians, 3 certified asthma educators, and 5 patients) for system testing. The mean System Usability Scale (SUS) score for the tool used in testing was 72.2 (SD 10.2). In the wiki stage, we recruited 41 participants (15/41 male) (9 pulmonologists, 6 primary care physicians, 5 certified asthma educators, and 21 patients) from diverse locations. The mean SUS score for the revised tool was 75.9 (SD 19.6). Users made 872, 466, and 599 successful changes to the AAP in weeks 1, 2, and 3, respectively. The site was used actively for a mean of 32.0 hours per week, of which 3.1 hours per week (9.7%) constituted synchronous multiuser use (2–4 users at the same time). Participants averaged 23 (SD 33) minutes of login time and made 7.7 (SD 15) changes to the AAP per day. Among participants, 28/35 (80%) were satisfied with the final AAP, and only 3/34 (9%) perceived interstakeholder group hierarchies.

**Conclusion:**

Use of a wiki-inspired method allowed for effective collaborative design of content and format aspects of an AAP while minimizing logistical requirements, maximizing geographical representation, and mitigating hierarchical group dynamics. Our method faced unique software and hardware challenges, and raises certain questions regarding its effect on group functioning. Potential uses of our method are broad, and further studies are required.

## Introduction

### Objective

We sought to develop and test an innovative wiki-inspired technology to facilitate collaborative design and consensus building across multiple stakeholders. In particular, our method aims to enable multiuser development of the content and format of media such as patient education tools. We developed and tested this technology through the collaborative design of an asthma self-management tool called an asthma action plan (AAP).

### Background And Significance 

An AAP is an individualized written plan produced by a health care professional (HCP) for a patient with asthma, for the purpose of providing education and guidelines for self-management of worsening asthma symptoms [1]. Because most AAP templates have been developed from a predominantly expert medical rather than from a primary care physician (PCP) or patient perspective [2], both AAP delivery by PCPs and AAP use by patients remains low [2,3], despite strong evidence for their benefit [1].

Development methods that integrate the perspectives of all relevant stakeholders have been shown to be more likely to yield products that are appropriate to the local context and that effectively meet the needs of end users [4]. Accordingly, stakeholder engagement in the development process is a key determinant of the implementability of products such as evidence summaries [5,6], guidelines [7], and patient tools [8]. Accordingly, we sought to develop a more readily implementable AAP through a design process that would enable inclusion of multiple stakeholders, including PCPs and patients. 

The three main formal consensus techniques used in health care are the Delphi method, the nominal group technique (NGT), and the consensus development conference [9]. The Delphi method consists of questionnaires mailed serially to stakeholders, interim feedback mailed to individuals regarding group preferences, and aggregation of responses according to explicit statistical principles [9,10]. The “online Delphi” technique applies the same principles; however, participants complete questionnaires electronically and can be linked through a central computer that continually updates and displays group preferences to individual participants [10,11]. In the NGT, participants are assembled face-to-face, each records his or her ideas independently, and these ideas are listed for the group and discussed with the help of a facilitator. Individual judgments and votes are recorded and aggregated statistically to derive the group judgment. Finally, a consensus development conference consists of a moderated, unstructured meeting where evidence and ideas are presented by various interest groups or experts who are not members of the decision-making group, and the latter retire to attempt to reach consensus. Both the open and the private group discussions are chaired, and implicit methods are used to aggregate opinions [9].

These techniques present several disadvantages for development of an AAP. First, an AAP is a visual medium. Previous studies have demonstrated the advantage of incorporating human factors design elements in visual media intended for health care interventions [12]. However, existing consensus and focus group techniques are poorly suited to achieving agreement about the physical attributes (format) of visual media [9] or for novel visual media design, due to inherent difficulties in expressing aesthetic preferences and describing imagined visual characteristics verbally [4]. Second, in-person techniques have a number of practical limitations. A skilled moderator is required [13,14], and may be difficult and expensive to access [6]. Other costs include travel and accommodation for stakeholders. Planning requires organizational support, and recruitment of appropriate participants can be challenging due to conflicting schedules [5,6]. Finally, in-person techniques are subject to complex group dynamics. Participants may be pressured to agree with a group’s or a dominant individual's viewpoint [9,15], and social hierarchies may form, favoring professionals over patients. Some individuals may not articulate their preferences due to a lack of confidence, a lack of trust in the group, or poor group management by the moderator [9,15].

We conceived of a novel wiki-inspired method to achieve both consensus and collaborative design. A wiki is a hypertext-based collaborative software that allows users not only to add content, but also to edit and alter existing content according to their preferences. Wikis have been used for collaborative writing, but not for development of visual media [16]. In medical research, wikis have been used to support the implementation of an electronic medical record system [17] and to build online catalogues of genetic codes, protein structure [18,19], medical ontology [20], and medical information [21,22]. Use of a wiki platform to ascertain and summarize the preferences of multiple users or in the design of a medical communication tool such as an AAP has not previously been reported.

## Methods

We developed and tested a system that allows multiple users to collaboratively design an AAP by inputting preferences for the content and format (visual layout and design) of the AAP through a Web-based wiki-inspired platform. In order to accurately reflect format permutations, users constructed and viewed the AAP in real time, as they navigated a series of choices in drop-down menus. All elements of the study were approved by our institutional review board.

### Development Stage

**Figure 1:**
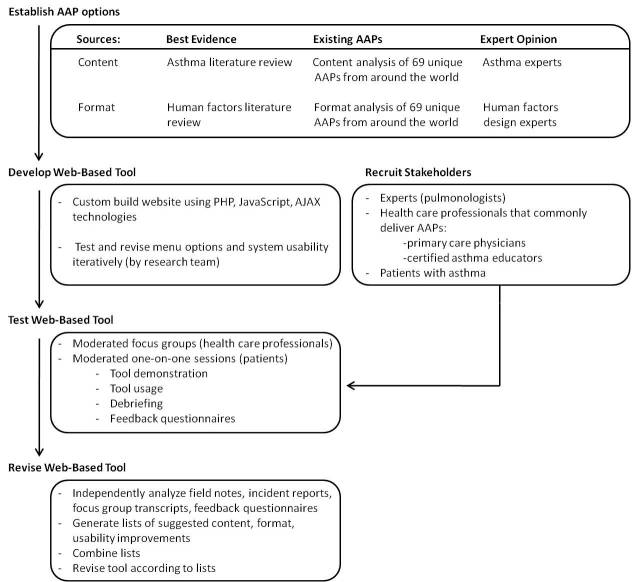
Method development process used in developing our wiki tool: (1) establish content and format options to include in the system, (2) build a Web-based application to enable representation of each content and format permutation through iterative revisions by the research team, while concurrently recruiting participants for the next stage, (3) test the tool in each stakeholder group to optimize content and format choices and system usability, (4) revise the tool based on data from the testing stage.

Development occurred in 4 steps (Figure 1). First, we established content and format options to include in the system, using best evidence from medical and human factors literature, a review of 69 existing AAPs collected from around the world, and opinions from asthma and human factors experts. Second, we built a Web-based application to enable representation of each content and format permutation (for the Safari Web browser, version 5.0.4; Apple Inc, Cupertino, CA, USA). Members of the research team serially tested and revised the system. Third, the tool was tested by stakeholders to optimize content and format choices and system usability. Relevant stakeholders were asthma experts (pulmonologists), the HCPs who commonly deliver AAPs (PCPs and certified asthma educators [CAEs]), and patients with asthma. Participants were purposively sampled to reflect hospital and community practice settings. Pulmonologists, CAEs, patients, and PCPs were recruited from a quaternary care hospital (St. Michael’s Hospital, Toronto, Ontario, Canada), and PCPs and patients were recruited from community clinics within the Greater Toronto Area. Patient inclusion criteria included a self-reported physician diagnosis of asthma, ability to speak English, and adequate computer skills (as determined by a brief screening questionnaire; please see the supplementary table in Multimedia Appendix 1).

We conducted 2-hour focus group sessions for HCPs (2–4 participants each) and 2-hour individual sessions for patients. Each session was facilitated by a moderator and attended by the study coordinator and computer programmer, who undertook troubleshooting, and by 2 study investigators, who took field notes. Sessions were audio recorded and the tapes transcribed verbatim. Each session was scripted and began with a presentation providing background on AAPs and the purpose of the study, followed by a tool demonstration and a 45-minute period during which participants were asked to individually use the tool to develop a “best possible” (blank) AAP. Study investigators observed each participant and documented difficulties on a standardized case report form. Copies of each participant’s final AAP were printed and distributed to all participants. In a group debriefing session, we discussed each case report to elucidate problems and corresponding improvements, and sought feedback on system usability and choices in each menu. Participants completed an online questionnaire consisting of a series of statements with 5-point Likert scales measuring agreement, open-ended questions, and the System Usability Scale (SUS) [23]. Participants were reimbursed for their time.

Finally, 2 members of the research team independently analyzed all field notes, case reports, focus group transcripts, and online feedback. Each member generated a list of suggested changes to content and format options and usability features of the tool. We revised the tool based on these suggestions.

### Wiki Stage

We used this revised tool for collaborative design in a wiki environment (tool available online at http://knowledgetranslation.ca/octapus_i/login.php?access=guest). This tool was inspired by the wiki concept and was similar to conventional wikis in the following ways: Web-based; used collaboratively by multiple users; invited all users to add edits; did not require any browser add-ons for core site functions; acted as a database for creating, browsing, and searching information; allowed for nonlinear, evolving, complex, and networked text, argument, and interaction; enabled real-time webpage creation and updating (without review before modifications were accepted and displayed online); and enabled a natural selection process to guide site content [24]. However, our technology also differed from conventional wikis in the following important ways: did not make use of simplified markup language or a “wysiwyg” text editor; did not invite casual users to be part of the wiki process; constrained edits by offering predetermined options rather than “free text” editing; offered users the ability to edit visual characteristics (format) of the website itself, rather than text content exclusively; and was not powered by wiki software (we used a custom-built platform rather than the MediaWiki software). Our application was custom built on the following frameworks: jQuery, version 1.3.1 (a JavaScript library with built-in AJAX functions was used for the client-side interaction); wkpdf, version 0.2 (used for PDF generation); PHP, version 5.2 (including PEAR and MDB2) (used for server-side functionality); and MySQL, version 5.1 (used for databases; Oracle Corporation, Redwood Shores, CA, USA).

We recruited 3 groups of new users, each composed of 14 participants (3 pulmonologists, 2 PCPs, 2 CAEs, and 7 patients with asthma) sampled purposively to reflect a broad range of settings. HCPs were recruited from hospitals and community clinics in Canada, from hospitals in the United States and Australia, from a Canadian AAP workshop, and through the Ontario Lung Association. Patients were recruited from hospitals and community clinics within the Greater Toronto Area and through the Asthma Society of Canada. Patients required a self-reported physician diagnosis of asthma, and all participants had to fulfill the requirements listed in the supplementary table in Multimedia Appendix 1 as well as the following: (1) access to high-speed Internet at work or at home, (2) average weekly Internet use at work or home of ≥4 hours, and (3) minimum once-weekly use of at least three of the following applications: email, Internet Explorer, Mozilla Firefox, Apple Safari, Microsoft Word, Microsoft Excel, Microsoft PowerPoint, or Adobe PDF.

We conducted orientation sessions 1 week before the start of each wiki session, summarizing AAPs, the study’s purpose, and the system’s functions. HCPs received this orientation through a 1-hour moderated Livestream webinar (Livestream, New York NY, USA) and patients through moderated face-to-face group sessions (2–4 participants each). Each user was asked to download Safari and to confirm the tool’s function on his or her computer before these sessions.

Each 14-participant group was given a 1-week period to collaboratively author a single AAP. Participants collaborated through the site’s wiki function, whereby any member could alter online choices made previously by other members. Participants received daily reminders to use the tool. The tool included a log of previously made choices, a chat room for online discussions, and comment fields attached to each choice, enabling members to propose supporting arguments for their choices. In the event of an ‘‘edit war” [16], defined as 14 serial changes to a single menu option made by 2 participants over a 48-hour period, the tool automatically triggered an online vote of all group members. The result from this vote would determine the option choice. Users had 24-hour access to the site and to technical support through email and telephone.

At the end of each 1-week period, participants received their group’s final AAP and completed an online questionnaire measuring perceptions of the tool, the wiki process, and the AAP. We documented logistical and technical difficulties associated with the technology and analyzed tool usage. We used expert opinion to define the following criteria for a successful wiki process: (1) high usage rates (a mean of ≥10 minutes of active logged-in time per user per day), (2) positive measures of usability (a higher mean SUS score than in the development stage, and a mean SUS score ≥72.5, corresponding to “good” or better usability) [25], and (3) high user satisfaction with the final product (a decreasing trend for changes made through the week-long process, and ≥75% user agreement with questionnaire statements relating to satisfaction with the final AAP).

## Results

### Development Stage

For testing, we recruited 16 participants (9/16 male) (4 pulmonologists, 4 PCPs, 3 CAEs, and 5 patients). Of the 16 participants, 7 (44%) were between 30 and 39, 4 (25%) were between 40 and 49, 4 were (25%) between 50 and 59, and 1 (6%) was ≥60 years of age. Of the 5 patients, 1 (20%) had a high school education, 1 (20%) had a college or trade school education, and 3 (60%) had a university education. The mean SUS was 72.2 (SD 10.2): 75.0 (SD 8.16) for pulmonologists; 76.2 (SD 11.1) for PCPs; 66.7 (SD 5.77) for CAEs; and 70.0 (SD 13.5) for patients (scores for the SUS can range from 0 to 100). We made several significant usability-related changes to the system on the basis of feedback received in the focus groups and interviews in the development stage. Table 1 presents user comments and corresponding revisions.

**Table 1:** Usability comments and corresponding improvements to the wiki-based asthma action plan (AAP) development system

Difficulty/criticism	Change made to system
Users ran out of space to add items to the AAP without realizing it	Added a pop-up warning for users when space was exceeded
	Added a hover above each layout to indicate approximate number of options that could be added in each AAP zone, with each layout
Users felt that their ability to experiment with different 3- vs 4-zone formats and a 1- vs 2-step yellow zone was limited by the fact that information added to the extra zone/step was lost if they switched formats	Reprogrammed the system such that items last contained in the orange zone or second step of the yellow zone would reappear if the zone/step was added back after having been removed
Users did not realize when a menu had ended and when to move to the next menu	Added a statement at the end of each menu section indicating that it is complete and directing users to the next appropriate section
Users did not find the use of arrows intuitive for opening and closing menus	Arrows were changed to “+” and “–” signs used in conventional Windows navigation
Users did not realize that choosing options in certain menus would automatically open further submenus containing phrases to complete these (otherwise nonsensical) statements	The following note was added below menu titles for all menus containing any such submenus: “*(note: selecting this option may produce further submenus with more options for you to choose)”*
When trying to remove or alter an existing item in the AAP, users often found it difficult to find the corresponding menu	Added a function such that double clicking on a selection in the AAP would open up the corresponding content root menu in the menu window
Users wanted to “line up” similar items across zones and control the order of items in each zone description and instruction area	Added a function such that items in each of the zone description and instruction areas could be reordered by clicking and dragging directly in the AAP window
Several users did not scroll down to see all menu options when menus were opening downward (these were hidden by the menu window box and required scrolling)	Reprogrammed menus such that each menu could be seen rising to the top of the menu window box once expanded, displaying scrollable choices below
Users indicated that they required more screen space to view the AAP (particularly laptop users)	Added a function enabling users to temporarily hide menus and to click and drag the entire AAP to the center of the screen for viewing
Users preferred to have similar titles for each zone and found it cumbersome to choose these separately in each zone menu	Added zone title selections to the “Setup” tab, such that these choices could be made consecutively

### Wiki Stage

We recruited 41 participants (15/41 male) (9 pulmonologists, 6 PCPs, 5 CAEs, and 21 patients) from 16 different cities, 5 Canadian provinces (Ontario, Quebec, Manitoba, Alberta, and British Columbia), and 3 countries (Canada, United States, and Australia). Although our target was 42 participants, 1 CAE who was recruited to the second wiki week withdrew, and we were unable to successfully recruit another participant in time for that week. This CAE later successfully participated in the third wiki week.

Among 39 participants whose age was available, 3 (8%) were <30, 10 (26%) were between 30 and 39, 14 (36%) were between 40 and 49, 9 (23%) were between 50 and 59, and 3 (8%) were ≥60 years of age. Of the 21 patients, 2 (10%) had a high school education, 10 (48%) had a college or trade school level education, and 9 (43%) had a university education. Our analysis focused on participation, system access, system usage, and user perceptions in the wiki stage.

#### Participation

Of the 42 target participants, 7 (17%) did not participate in the process: 3 missed the training seminar, due to a family emergency (1), sudden illness (1), and inability to access Livestream due to university firewalls (1); 2 could not download Safari due to university firewalls; 1 did not register for the site after training; and 1 did not log in despite technical assistance. Of the 42 target participants, 5 (12%) reported reasons for limited participation: 2 could not download Safari to an office computer due to firewalls but accessed the site from home; 1 had computer problems that limited participation; 1 could not download Safari to an office computer and had problems on the home computer, and 1 was hospitalized for 3 days of the 7-day wiki process. Lost and limited participants were approximately evenly distributed across wiki weeks and participant types, and all 3 wiki weeks had full-time participation from at least one of each user type.

#### System Usage

Of 347 login attempts over the 3 wiki weeks, 128 (36.9%) failed due to use of incorrect browsers. With the help of technical support personnel (mostly through email communication), all but 1 participant eventually successfully accessed the site through Safari. A total of 872, 466, and 599 successful changes to the AAP were made in wiki weeks 1, 2, and 3, respectively. Of these 1937 changes, 453 (23.4%) related to AAP format and 1484 (76.6%) to content, and no edit wars occurred. A video demonstrating the evolution of the AAP over the first wiki week is available in Multimedia Appendix 2. One PCP (week 2), and 1 pulmonologist (week 3) logged in but did not make changes to the AAP. The mean number of conversations (≥2 participants exchanging chat messages) was 8.0/week, with an average of 5.8 messages and 2.8 participants per conversation. Through comments and chats, 6 of 19 (32%) patients and 7 of 17 (41%) HCPs (total 13/36, 36%) revealed the stakeholder group to which they belonged. The site was used actively for a mean of 32.0 hours per week, of which 3.1 hours/week (9.7%) constituted synchronous multiuser use (2–4 users at the same time). Table 2 details the system usage data.

**Table 2:** System usage data

	Participant type				
	Pulmonologists	Primary care physicians	Certified asthma educators	Patients with asthma	All users
Mean (SD) number of days logged in/week/user	3.1 (2.0)	4.4 (1.5)	5.2 (1.6)	5.3 (2.0)	4.7 (2.0)
Mean (SD) number of logins/week/user	4.4 (5.8)	5.8 (5.2)	6.4 (6.2)	6.7 (6.6)	6.1 (6.3)
Mean (SD) active logged-in time/day/user^a^ (minutes)	14 (24)	16 (23)	38 (50)	24 (31)	23 (33)
Mean (SD) number of changes made/day/user	6.3 (14)	6.0 (12)	7.2 (9.9)	8.8 (17)	7.7 (15)
Range of total number of changes made/user (in 1 week)	0–148	0–146	10–102	5–357	0–357
Mean (SD) number of changes made/login	9.9 (21)	7.7 (13)	8.0 (10)	9.3 (17)	9.0 (16)
Mean (SD) number of comments posted/user (in 1 week)^b^	3.4 (3.9)	2.0 (2.4)	5.4 (10)	0.9 (1.8)	2.2 (4.5)
Mean (SD) number of chat entries/user (in 1 week)^b^	3.9 (5.3)	5.6 (6.1)	8.8 (9.3)	4.0 (4.4)	5.0 (6.0)

^a^ The website did not have an automatic time-out feature, as we wanted to encourage users to keep it open on their desktops for periodic daily access. Given that some users remained logged on for a number of hours at a time, in order to estimate accurate “active” usage times, we truncated all logged-in times at 30 minutes after the last “activity” (including any change made, or comment or blog posted). In cases where two activities were separated by >60 minutes, we truncated logged-in times at 30 minutes after the first activity and 30 minutes before the second activity (for a maximum total “active” usage time of 60 minutes between activities).

^b^ Chat and comment features were used by 28/35 (80%) and 16/35 (46%) participants, respectively.

**Figure 2:**
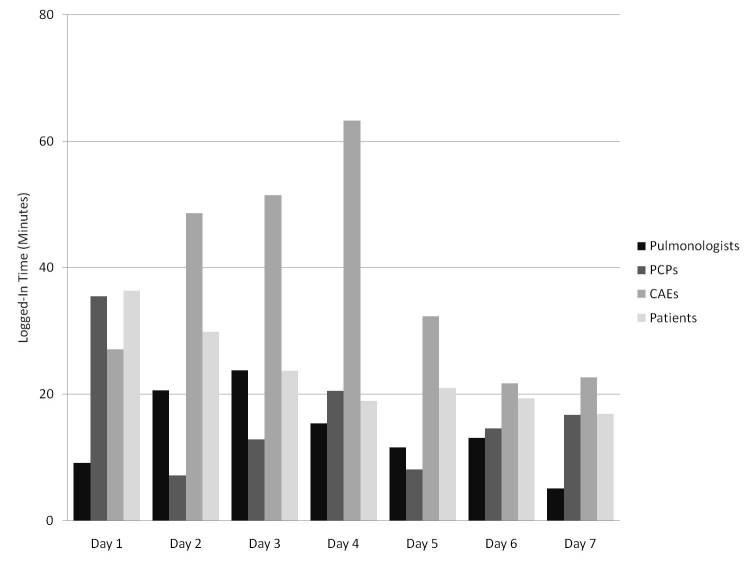
Mean logged-in time per participant per day, by participant type (CAE = certified asthma educator; PCP = primary care physician).

**Figure 3:**
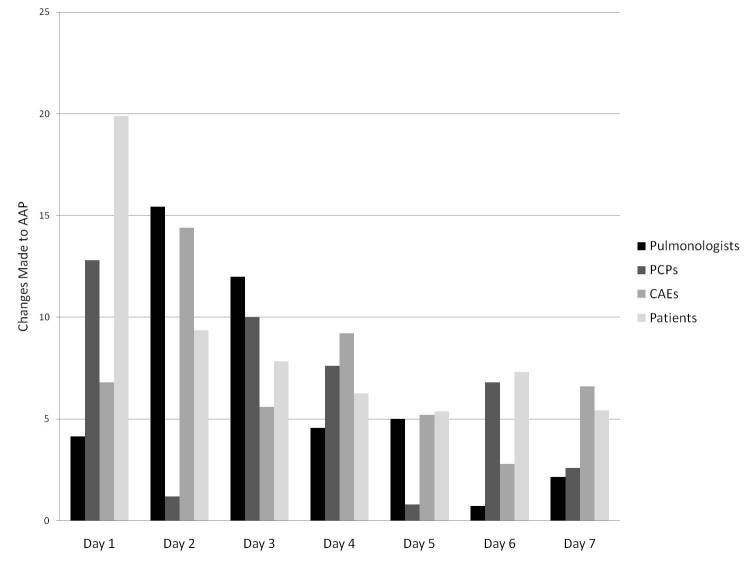
Mean changes made to the wiki-based asthma action plan (AAP) template per participant per day, by participant type (CAE = certified asthma educator; PCP = primary care physician).

**Figure 4:**
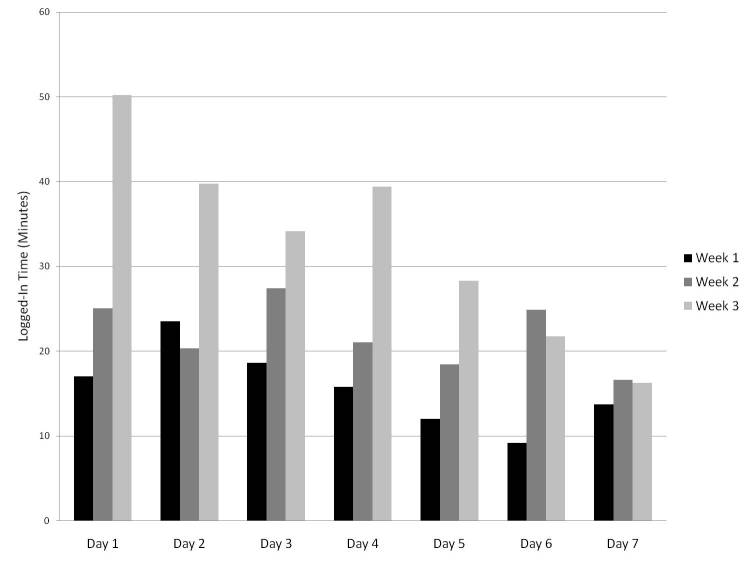
Mean logged-in time per participant per day, by week.

**Figure 5:**
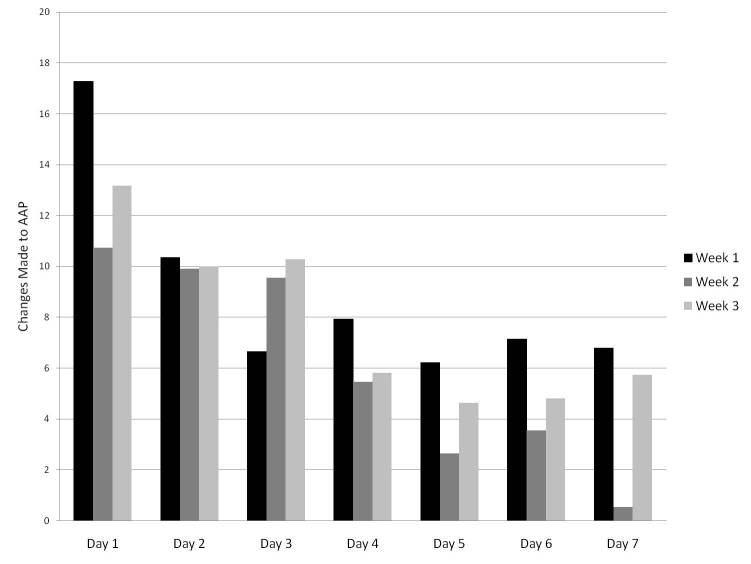
Mean changes made to the wiki-based asthma action plan (AAP) template per participant per day, by week.

Temporal trends for site usage and for changes made (by user type) are shown in Figure 2 and Figure 3, respectively. In Figure 2, each bar represents the mean number of minutes that each user was logged in to the system on each day, by user type, and by day of the wiki process. In Figure 3, each bar represents the mean number of changes that each user made to the AAP on each day, by user type, and by day of the wiki process. In both figures, averages were based on all users who had access to the site, and data from all 3 wiki weeks were averaged. A similar decreasing trend for usage (Figure 4) and changes made throughout the week (Figure 5) was seen in each of the 3 wiki weeks. In Figure 4, each bar represents the mean number of minutes that each user was logged in to the system on each day, by wiki week, and by day of the wiki process. In Figure 5, each bar represents the mean number of changes that each user made to the AAP on each day, by wiki week, and by day of the wiki process. In both figures, averages were based on all users who had access to the site, and data from all participants were averaged.

The final AAP had 153 of 229 (67%) choices in common with the AAP at the end of the first day, and 108 of 229 (47%) of the choices that had been made by the end of the first day were not changed through the rest of the week. A detailed description of the final AAP will be published elsewhere (S Gupta, MSc, MD, et al, unpublished data, 2011).

#### User Perceptions

**Figure 6:**
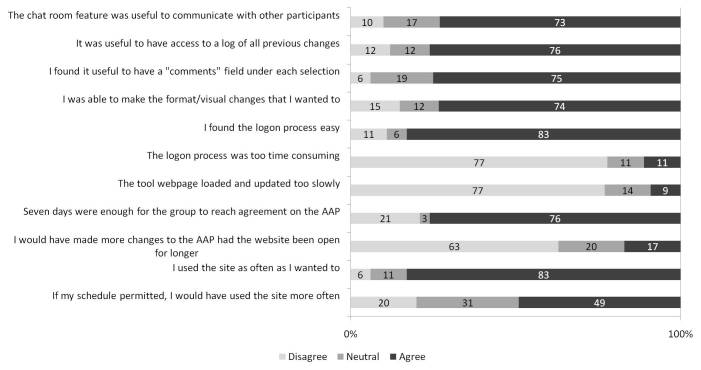
Specific features of the wiki tool and wiki process. Responses were entered on a 5-point Likert scale labeled as follows: 1, disagree; 3, neutral; and 5, agree. For the purposes of this figure, scores of 1 and 2 were considered “disagree,” and 4 and 5 were considered “agree.” Each bar demonstrates the proportion of participants with each response, for each statement. This includes 35 participants (5 certified asthma educators, 5 primary care physicians, 6 pulmonologists, and 19 patients with asthma). (AAP = asthma action plan).

**Figure 7:**
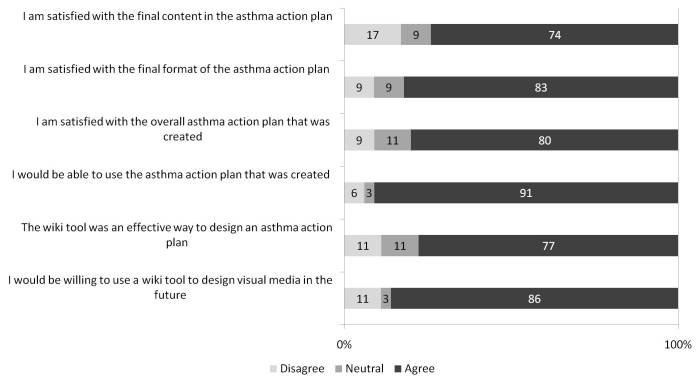
Overall asthma action plan (AAP) and the wiki process. Responses were entered on a 5-point Likert scale labeled as follows: 1, disagree; 3, neutral; and 5, agree. For the purposes of this figure, scores of 1 and 2 were considered “disagree,” and 4 and 5 were considered “agree.” Each bar demonstrates the proportion of participants with each response, for each statement. This includes 35 participants (5 certified asthma educators, 5 primary care physicians, 6 pulmonologists, and 19 patients with asthma).

**Figure 8:**
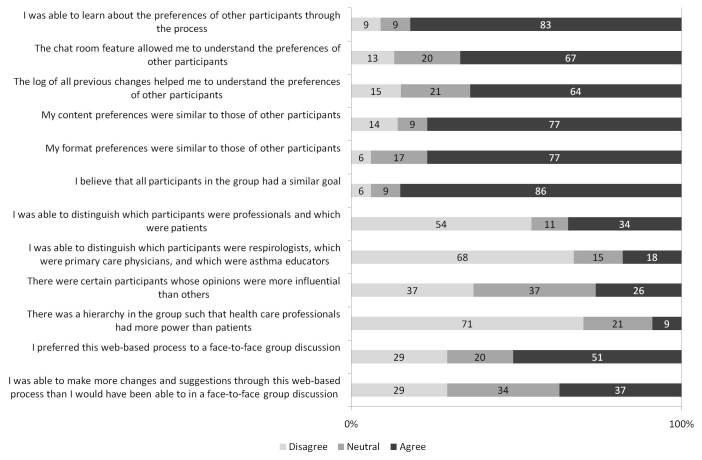
Participant interactions. Responses were entered on a 5-point Likert scale labeled as follows: 1, disagree; 3, neutral; and 5, agree. For the purposes of this figure, scores of 1 and 2 were considered “disagree,” and 4 and 5 were considered “agree.” Each bar demonstrates the proportion of participants with each response, for each statement. This includes 35 participants (5 certified asthma educators, 5 primary care physicians, 6 pulmonologists, and 19 patients with asthma).

Participant Likert scale responses are summarized in Figure 6, Figure 7, and Figure 8. Of 25 participants, 11 (44%) indicated that they changed their minds about one or more issue(s) based on other participants’ preferences.

Reported barriers to tool use included time constraints, difficulties with the login process, no access over work networks, and software bugs. Additional challenges included redundant choices, limited content choices, “information overload,” limited amount of space in the AAP, small size of the comment box (requiring frequent scrolling), lack of participant accountability for changes made, difficulty explaining one’s point of view through an online chat, and technical challenges understanding site functions. Reported advantages of the system included tool accessibility, broad recruitment, the wide range of available format and content options, tool responsiveness, ability to communicate with peers, and power balance between users, enabling participation by “shy” and “insecure” participants, and averting potentially unpleasant social dynamics. The mean SUS score was 75.9 (SD 19.6).

## Discussion

We developed a novel technique to achieve multiple stakeholder consensus and design, and tested it in the development of an AAP.

### Principal Results

Nearly all measures exceeded our expert criteria for an effective technique. Most participants used the tool daily, and each user type actively used the tool for an average of 23 minutes per day. The mean SUS score of 75.9 (SD 19.6) was higher than that in the development stage, indicating system improvement. This score falls in the third quartile of usability scores for other types of tools, indicating good to excellent usability, and is within the range associated with a high chance of real-world user acceptability [25,26]. The data from the SUS can be triangulated with data from the exit questionnaires, which demonstrated greater than 70% user agreement with all usability-related statements, indicating that participants favored its usability highly. Finally, exit questionnaires demonstrated 75% or greater user agreement with nearly all statements relating to satisfaction with the final AAP.

### Comparison With Existing Techniques

Conventional focus group or consensus techniques provide limited ability both to measure preferences for and to develop consensus around document aesthetics [4]. Our method has met this challenge; 23.4% (453/1937) of changes related to the format of the AAP, 74% (26/35) of users were able to make the format or visual changes that they wanted to, and 83% (29/53) of participants were satisfied with the AAP’s final format. Our process also fulfils the recommended criteria for media design: (1) suitability for all stages of the design process (which can be achieved through iterative wiki design stages), (2) flexibility to adapt to the varying requirements of the design process (which can be achieved through iterative changes to options offered in the wiki tool), and (3) presentation of visual information in a format that inspires users [4]. A caveat is that options in the wiki site must be predetermined, possibly limiting user creativity [4]. An alternative would be to offer users the ability to enter “free text” for tool content, such as in a conventional wiki, although this approach could not easily be applied to format options. 

Our method was logistically simpler than other techniques for achieving consensus. Although we used moderators in the development process and in training sessions, unlike in the NGT, consensus development conference, and focus groups, the wiki process does not require a moderator. This method eliminates the task of finding a qualified moderator and associated costs [6], and averts potential pitfalls including poor facilitation, undue influence on participants, and minimization of certain participants’ views [27]. A caveat is that site usage was variable and 2 users did not make any changes after logging in; moderators could serve to encourage both universal and more equal participation. 

By eliminating the need for participants to meet, we limited organizational and recruitment challenges, and costs incurred with in-person consensus techniques. Our method also enabled international representation, minimizing the geographical bias seen with in-person techniques, and at no incremental cost [9,13]. Another advantage to this method is that preferences are not fixed over time, and attitude change and idea generation may have been enhanced by the 7-day period for interaction, compared with a conventional single in-person meeting [15]. In-person techniques are usually limited to between 5 and 10 participants due to difficulty in coordinating schedules, cost of accommodation, and concerns about group function, with larger sizes favoring unequal participation [9,27]. Our method successfully accommodated groups of 14 participants. Although there is little empirical evidence regarding the effect of the number of participants on the validity of consensus processes, larger groups likely increase the reliability of group judgment [9].

Finally, our method addressed certain challenges arising from complex group dynamics experienced with in-person techniques. Social impact theory suggests that group judgments have a strong influence on individual decision making [9]. In face-to-face consensus processes, participants often define subgroups by stakeholder types. They may be pressured to conform to their own group, and consensus building can be inhibited by intergroup prejudices and stereotypes [9]. Furthermore, the status of individual participants affects their influence on other participants, and status hierarchies—both between professionals and patients, and within professional groups—are likely to emerge in face-to-face meetings [9,28]. These hierarchical structures limit the willingness of some participants to contribute openly [13]. The wiki method minimizes any group or individual influence by anonymizing stakeholders and by eliminating verbal communication, which is both a source and an indicator of status within groups [28]. Although certain participant group identities were revealed through chat and comment entries, only 9% (3/35) of participants perceived a power differential between users. Overall, most participants reported successfully learning about the preferences of others and believed that everyone had a similar goal; 37% (13/35) were also able to make contributions that they felt they could not have made in a face-to-face forum. However, it is possible that HCPs prefer the hierarchical power differential that they enjoy in face-to-face forums. This may partly explain the dichotomous responses to whether participants preferred the Web-based process to a face-to-face discussion, and whether they felt that the Web-based process enabled them to effectively make more changes (Figure 8).

The Delphi method shares the resource, time, and recruitment advantages of the wiki method and avoids concerns related to group functioning. However, in contrast to the wiki method, the Delphi method does not capture the important synergistic effect of participant interaction on the development of ideas [4], lacks a mechanism for conflict resolution in areas of disagreement (enabled by chat room discussions, comment fields, and the online vote feature in the wiki method), and does not identify the reasons for disagreements (enabled by qualitative analysis of chat room discussions and comment fields in the wiki method) [9]. The online Delphi method shares the same platform as the wiki method (the Internet) and has been shown to be more time and cost efficient than the traditional Delphi method [10,11], but shares the limitations described above. In addition, Web content is limited to text, and this method does not enable measurement of group preferences or group collaborative development around document aesthetics [10].

### Limitations

We noted some logistical and technical difficulties. A moderate proportion of the targeted sample had no or limited participation, in some cases due to unexpected changes in personal circumstances, but in most cases due to software access difficulties caused by firewalls. Although full-time representation from each user type was maintained in all 3 wiki weeks in this study, differential dropout rates between user types can threaten the validity of the process and should be addressed in future studies. Researchers should anticipate such losses in setting recruitment targets, instruct users to test all required websites on all computers that they intend to use during the process, and verify site functionality with users before the process begins. 

Users also struggled with software instructions, as demonstrated by the large number of failed logins, likely because Safari was not their default browser. This did not have a significant impact on usage, as all but 1 participant successfully logged on. However, this demonstrated the importance of 24-hour technical support in enabling this process. Future studies of the wiki method should emphasize use of the correct browser at the orientation stage and consider building cross-browser compatibility. 

Although the wiki process averted the costs of hiring a moderator and organizational and travel costs incurred in in-person techniques, development process costs were considerable. These include costs for software development, moderated in-person tool testing, and tool revisions based on user feedback. Technical support and analysis of wiki session data are additional costs.

There are several caveats regarding the effect of the wiki process on group functioning. Attitude change is an important part of consensus building [9]. This requires persuasive ability, which can be influenced by visual cues (eg, facial expressions), and paralinguistic cues (eg, voice quality), and depends on the credibility, trustworthiness, and likeability of the communicator, all of which may be better transmitted through in-person interactions [9]. Elements such as tone of voice, and facial and body expressions are useful “human” cues that are lost in the wiki method [4]. Although the effect of online communication (either synchronous or asynchronous) on individual decision making is unknown, 44% (11/25) of participants indicated that they changed their minds about one or more issue(s) based on the preferences of their peers. However, a minority believed that they were able to make more changes in the Web-based tool than they could have in a face-to-face forum. This may also relate to the fact that user options were limited to those offered in drop-down menus, as opposed to a theoretically unlimited number of options available in a face-to-face discussion.

Motivation to participate in this process may be similar to motivations that have made Wikipedia one of the most visited Internet sites in the world—the pleasure, validation, and sense of ownership that users derive from seeing their personal edits and contributions [16]. Although overall user engagement was strong, 2 users did not make any changes. This “social loafing,” whereby certain group members leave the bulk of the work to others [9], may be exacerbated by the anonymity in the wiki process, compared with face-to-face processes, where social loafers risk embarrassment. Anonymity might also facilitate a contrarian or destructive contribution pattern, and although this is unlikely in a carefully recruited group, lack of accountability was cited as a disadvantage by 1 user.

Organizational psychological research suggests that participants’ initially expressed opinions may disproportionately influence consensus group decisions [9]. The wiki process is particularly susceptible to this bias, as it begins with a “blank slate” that the first few participants alter to create the first recognizable form of the tool, which is then edited by others. We noted that 67% (153/229) of the choices in the final AAP had been made at the end of the first day, and 47% (108/229) of the choices that had been made at the end of the first day were never changed throughout the week. Conversely, the wiki process is susceptible to a single participant or a small group of participants making substantial changes just before the process end time, threatening the collaborative or consensus nature of the outcome. Remarkably, this did not occur in any of the 3 wiki weeks. Furthermore, as Figure 3 shows, the mean number of changes made per day trended downward as the week progressed, with the fewest changes made in the last 3 days. This may reflect progressively increasing user satisfaction with the developed product. 

### Conclusions

We developed a unique wiki-inspired process for collaborative design and consensus building and applied it in the development of an AAP. The name “WikiBuild” was chosen to capture the essence of this technology—a wiki-inspired tool designed to enable users to collaboratively build a multitude of different products. Potential uses of our method are broad, and include development of both medical and nonmedical tools and products. Commercial uses may include development of marketing material by members of the target consumer group itself, or codevelopment by designers and target consumers. Future studies should address software- and hardware-related technical challenges and questions about the dynamics and functioning of virtual focus groups. Novel variations of our study design should also be considered, such as testing larger wiki groups, or running separate wiki processes in different stakeholder types in order to explore differences between stakeholder preferences.

## Multimedia Appendix 1

Development stage inclusion criteria.

## Multimedia Appendix 2

Video demonstrating the evolution of the AAP over the first wiki week.

## Abbreviations

AAP: asthma action plan
CAE: certified asthma educator
HCP: health care professional
NGT: nominal group technique
PCP: primary care physician
SUS: System Usability Scale
