# Supplementary material for: WikiBuild: A New Online Collaboration Process For Multistakeholder Tool Development and Consensus Building
Source: J Med Internet Res. 2011 Dec 8;13(4):e108. doi: 10.2196/jmir.1833 (PMC3278094; doi:10.2196/jmir.1833)
Supplement: Supplementary file 1 [file jmir_v13i4e108_app1.pdf]

Supplementary Table 1. Development Stage Inclusion Criteria

**Question***	**Accepted Response Range┼**
I use computers only because they are necessary for work	Disagree or Strongly Disagree
Computers have a positive impact on my quality of life	Agree or Strongly Agree
I find dealing with computers to be frustrating	Disagree or Strongly Disagree
I am confident in my ability to master new skills with computers	Agree or Strongly Agree

* Participants were asked to rate their agreement with each statement on a scale of 1 to 5, with 1 indicating strongly disagree, 5 indicating strongly agree, and 3 indicating a neutral response

┼ Responses to all 4 questions had to be in the accepted range for patients to be included in the study
